# Spondyloocular Syndrome: A Report of an Additional Family and Phenotypic Spectrum Delineation

**DOI:** 10.3390/genes14020497

**Published:** 2023-02-15

**Authors:** Eliane Chouery, Rim Karam, Yves Najm Mrad, Cybel Mehawej, Nahia Dib El Jalbout, Jamal Bleik, Daniel Mahfoud, Andre Megarbane

**Affiliations:** 1 Department of Human Genetics, Gilbert and Rose-Marie Chagoury School of Medicine, Lebanese American University, Byblos P.O. Box 13-5053, Lebanon; 2 Gilbert and Rose-Marie Chagoury School of Medicine, Lebanese American University, Byblos P.O. Box 13-5053, Lebanon; 3 Department of Ophthalmology, Lebanese American University Medical Center, Rizk Hospital, Beirut P.O. Box 13-5053, Lebanon; 4 Department of Radiology, Gilbert and Rose-Marie Ghagoury School of Medicine, Lebanese American University, Byblos P.O. Box 13-5053, Lebanon; 5 Institut Jérôme Lejeune, 75015 Paris, France

**Keywords:** spondyloocular syndrome, *XYLT2*, skeletal disorders, ocular disorders, whole exome sequencing, Lebanon

## Abstract

Spondyloocular syndrome (SOS, OMIM # 605822) is a rare genetic disorder characterized by osseous and ocular manifestations, including generalized osteoporosis, multiple long bones fractures, platyspondyly, dense cataracts and retinal detachment, and dysmorphic facial features, with or without short stature, cardiopathy, hearing impairment, and intellectual disability. Biallelic mutations in the *XYLT2* gene (OMIM * 608125), encoding the xylosyltransferase II, were shown to be responsible for this disease. To date, 22 cases with SOS have been described, with varying clinical presentations and a yet-to-be-established genotypic–phenotypic correlation. Two patients from a consanguineous Lebanese family that presented with SOS were included in this study. Whole exome sequencing revealed a novel homozygous nonsense mutation in *XYLT2* (p.Tyr414*) in these patients. We review all previously reported cases with SOS, describe the second nonsense mutation in *XYLT2*, and contribute to a better delineation of the phenotypic spectrum of the disease.

## 1. Introduction

Mendelian bone fragility disorders (MBFD) are a group of genetic disorders that have a detrimental effect on bone mineral density, leading to recurrent fractures in affected patients. These diseases can be inherited in an autosomal recessive, autosomal dominant, or X-linked manner. Osteogenesis imperfecta type 1 is the most recognized and most frequent form of MBDF (OMIM # 166200), but many other entities have been described depending on the age of onset, the severity of bone fractures, and the presence of non skeletal manifestations such as neurodevelopmental delay, dysmorphic features, or various organ system defects such as occular abnormalities.

Spondyloocular syndrome (SOS) (OMIM # 605822) is a rare autosomal recessive skeletal and ocular disorder, first described in 2001 by Schmidt et al. [1]. It is characterized by generalized osteoporosis leading to multiple fractures, vertebral flattening, and ocular abnormalities such as cataract, crystalline lens malformation, and retinal detachment. In addition, patients with SOS suffer from other characteristic morphological findings that are present to varying degrees in affected individuals, such as short stature, dysmorphic facial features, shield chest, genitourinary and cardiac malformations, and hearing impairment. In 2015, pathogenic mutations in the *XYLT2* gene (OMIM * 608125), located on chromosome 17q21.33, were found to be responsible for this syndrome [2]. *XYLT2* encodes an isoform of xylotransferase, belonging to the family of glycosyltransferases and involved in the biosynthesis of glycosaminoglycan chains (GAGs) in the proteoglycans (PGs). GAGs, a main component of the extracellular matrix, are important for the development and maintenance of bone, cartilage, skin, and other tissues [3]. PGs are composed of a core protein with hydrophilic GAGs that are O-linked to serine residues by a tetrasaccharide linkage region (Xyl–Gal–Gal–GlcUA). XYLT1/2 enzymes initiate the linkage step and then the process of elongation of the glycan linker is completed by three remaining enzymes, encoded by *B4GALT7*, *B3GALT6*, and *B3GAT3*, that add the three remaining sugars to complete a four-sugar linker [4]. Finally, a fifth sugar residue is added to determine the type of GAG being synthesized. PGs are ubiquitously expressed yet functionally diverse, playing several cardinal roles in signaling and cellular processing [4,5]. Any defect in any of the PG synthesis steps can thus lead to a multisystemic disorder. Linkeropathies are, for instance, multisystem hereditary connective disorders that are caused by enzymatic defects in the synthesis of the common linker region that joins the core proteins to their GAGs [6].

To date, a total of 22 cases with *XYLT2* mutations have been described, with varying clinical presentations and a yet-to-be-established genotypic–phenotypic correlation. We report on two additional cases from a consanguineous Lebanese family, describe the second nonsense mutation reported in SOS, review the literature, and expand the clinical spectrum of the disease.

## 2. Material and Methods

### 2.1. Patients

**Figure 1:**
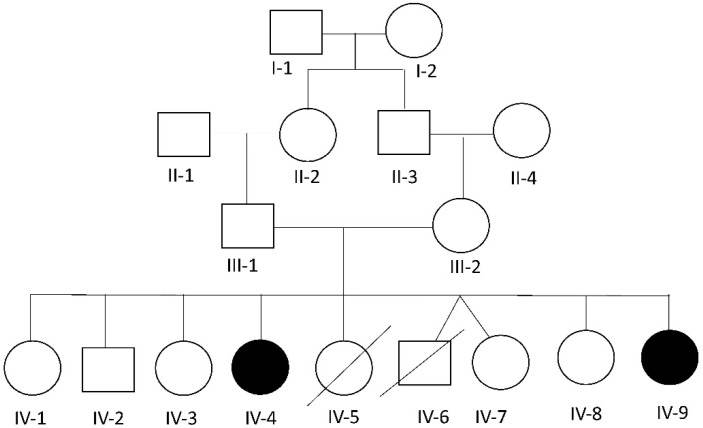
Pedigree of the reported family. Black color represents the affected individuals. Arrows indicate the affected individuals included in this study.

We herein describe a consanguineous Lebanese family (Figure 1) with two daughters (IV-4 and IV-9) affected with skeletal and ocular abnormalities that visited our clinic seeking clinical genetic diagnosis. Written informed consent was obtained from the parents of the patients (parental consent) to participate in this study, and approval was given for the publication of the findings.

### 2.2. Isolation of Genomic DNA

EDTA blood samples from all members of the family were collected for genetic studies. DNA was extracted from leucocytes by standard salt-precipitation methods as previously reported [7].

### 2.3. Whole Exome Sequencing (WES)

Whole exome sequencing (WES) was carried out in the patient. The exome was captured and enriched using the solution Agilent SureSelect Human All Exon kit version 5.0, and samples were then multiplexed and subjected to sequencing on an Illumina HiSeq 2500 PE100-125. Reads files (FASTQ) were generated from the sequencing platform via the manufacturer’s proprietary software. Reads were aligned to the hg19/b37 reference genome using the Burrows–Wheeler Aligner (BWA) package version 0.7.11 [8]. Variant calling was subsequently performed using the Genome Analysis Tool Kit (GATK) version 3.3 [9]. Variants were called using high stringency settings and annotated with VarAFT software 1.61 [10] containing information from dbSNP147 and the Genome Aggregation database (gnomAD, http://gnomad.broadinstitute.org accessed on 1 September 2021). Only nonsynonymous coding and splicing variants found in the patient were considered. Variant filtering was then performed based on the frequency of the variant in the gnomAD database (<0.01% and <50 heterozygous carriers or <5 homo-/hemizygous carriers), and in our in-house database (<1 homozygous occurrence), whereby only rare variants were selected. Additional filtering was carried out according to the mode of inheritance of the disease in the family. Indeed, considering the recessive inheritance of the disease in the pedigree and consanguinity, we assumed identity by descent and thus selected only homozygous variants.

## 3. Results

### 3.1. Clinical Presentations

Two siblings from a consanguineous Lebanese family presenting with skeletal and ocular abnormalities were investigated. For both, delivery was normal, with no recall of exposure to pre- or perinatal environmental toxins.

Patient 1: The first affected girl, IV-4 (Figure 1), is the fourth child in the family and was born at term from a 28-year-old mother and a 32-year-old father. At birth, her length was 53 cm (+0.7 SD) and her weight was 4000 g (+1.3 SD). Major complaints noted by the parents during her early development included recurrent episodes of constipation, bad urine smell, growth retardation, and developmental delay. The patient started walking without help at the age of 2 and a half years, although with some stepping, and talked more or less clearly by age 3. At around age 3, fractures happened recurrently after minor traumas, treated accordingly by cast and pins. At age 4, the patient received bisphosphonates treatment through pamidronate infusions, which improved bone mineral density and fracture frequency without reshaping the compression fractures. The latter treatment was discontinued by the parents after 5 months due to the limited availability of the drug in the local market, especially since the fracture recurrence had been reduced, albeit not completely resolved.

At the age of 6, a bilateral cataract was found during a routine ophthalmological examination and treated by cataract extraction with posterior chamber intraocular lens implantation and anterior vitrectomy in both eyes (OU).

**Figure 2:**
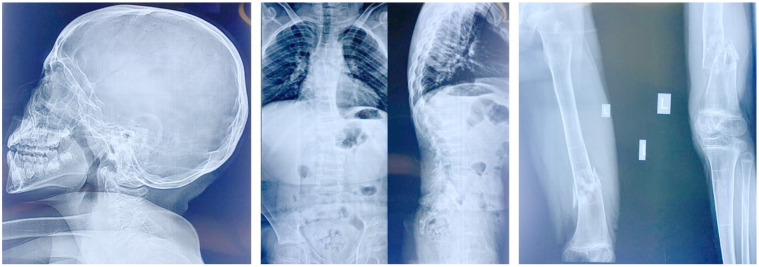
X-rays of patient 1 showing thinning of the occipital bone, abnormal vertebrae, kyphoscoliosis, femoral fractures, and bone demineralization.

When first seen by us at the age of 12 years, the patient was cooperative and could understand orders. Moderate intellectual disability was observed. Her head circumference (OFC) was 53 cm (0 SD), her height 126.5 cm (<−2.3 SD), and her weight 28 kg (<−2.3 SD). She had long palpebral fissures with long eyelashes; translucent teeth; thin lips; short and webbed neck; short trunk; kyphoscoliosis; increased inter-nipples distance; pectus carinatum; truncal obesity; flat feet; joint laxity; and one small café-au-lait spot on her lower back (Supplementary Table S1). External genitalia exam was normal with no pubertal signs. Visual acuity could not be assessed. Eye examination revealed the presence of thin cornea. Color vision by Ishihara plates was normal OU. Pupils were equal, round, and reactive with no afferent pupillary defect OU. Intraocular pressure was normal (13 mmHg) OU. Cover-uncover test showed a 20 prism diopters exotropia at near and distance with no pattern, alternating fixation, and horizontal nystagmus. Ductions and versions showed limited elevation, depression, and adduction of the right eye (OD), and full motility in the left eye (OS). Anterior segment exam showed a clear conjunctiva, white sclera, clear thin corneas (central corneal thickness of 346 µm OD and 343 µm OS; average central corneal thickness for age 343 µm is around 555 µm) with normal horizontal diameter (white-to-white, 10.87 mm OD and 11.83 Mm OS), deep and quiet anterior chamber OU, normal irides, PCIOL in place OU. Funduscopic exam showed a clear vitreous OU, optic discs with large cups but healthy rims, normal vasculature, and normal macula and periphery. Neurological and cardiac evaluations were unremarkable. A brain MRI was performed but did not yield any remarkable findings. A review of a skeletal survey performed throughout the years to assess the recurrent fractures revealed thinning of the occipital bone; multilevel flattening and biconcave compression of the vertebra mostly flattened at L1/L5; a scoliosis; a left coxa valga; a non-displaced transverse fracture of the mid diaphysis of the left radius and ulna that was stabilized with pins; healed spiral fractures of the mid third of the diaphysis on the left and right femurs with residual angulation and mild bowing of the left femur; comminuted fracture of the distal third of the diaphysis of the left femur with displacement; and bone demineralization and horizontal sclerotic stripes on both tibia and fibula (Figure 2). Assessment of bone density showed a Z-score of −4.88.

**Figure 3:**
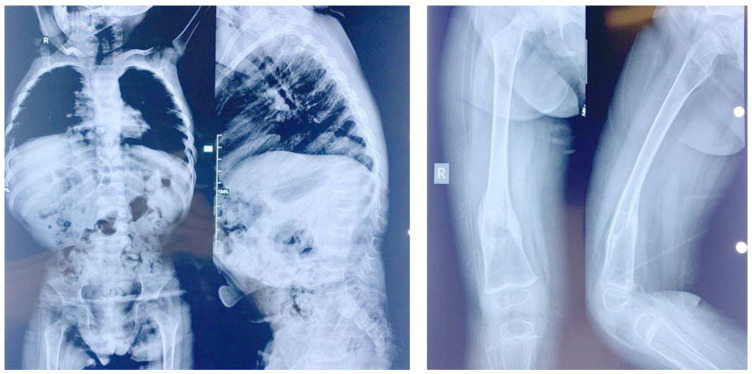
X-rays of patient 2. Note the abnormal lower dorsal and lumbar vertebra and the femoral fracture.

Patient 2: Subject 2 (IV-9 in Figure 1), a girl born 7 years after her affected sister, was seen at the age of 5 years. At birth, her length was 47 cm (−1.6 SD), and her weight was 3300 g (O SD). The parents noticed the same manifestations as her sister, including developmental delay, constipation, bad urine smell, and multiple fractures secondary to minor traumas in addition to a more prominent pectus carinatum. At the time of her examination, her OFC was 51 cm (O SD), her length was 83 cm (<−2.3 SD), and her weight was 11 Kg (−2 SD). She had the same clinical manifestations as her affected sister, in addition to a tide tongue and hyperextensible hip joints (Supplementary Table S1). External genitalia, neurological and heart examinations, as well as echocardiography and brain MRI were unremarkable. Ophthalmological examination revealed the presence of bilateral cataract with horizontal nystagmus. Analysis of her X-rays showed the presence of horizontal sclerotic stripes related to mineralization disorders; flattening of the vertebral body of the lower dorsal and lumbar vertebra; anterior wedge compression fracture of L1; biconcave flattening of L3; a healed subacute angulated fracture of the distal third of the left femoral diaphysis with sclerotic borders; a per-trochanteric impacted fracture of the left femoral neck; another angulated fracture of the distal diaphysis at the junction with the metaphysis; and a healed spiral fracture of the distal third of the diaphysis with bone remodeling (Figure 3).

Examination of the parents and their non-affected children showed a complete absence of all the features present in the affected siblings. A detailed family history revealed that two children (IV-5 and IV-6) died shortly after birth without any identified medical reason according to the parents.

### 3.2. Genetic Studies

**Figure 4:**
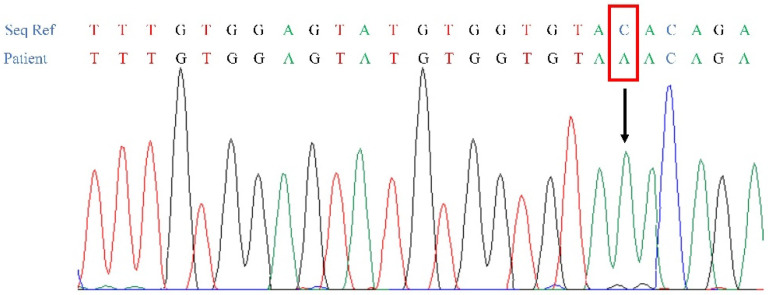
Electrophoregrams showing the identified p.Tyr414* (c.1242C>A) variant in *XYLT2* (NM_022167.3), in the affected patient (IV-4).

WES was performed in the patient IV-4 and led to the identification of approximately 97,866 variants. Considering the recessive inheritance of the disease in the pedigree and consanguinity, we assumed identity by descent and thus selected homozygous variants. This led to the selection of 36,903 variants. Additional filtering was then performed to exclude all non-genic, non-splice site, and intronic variants as well as all frequent variants (present in more than 1% in databases). This filtering strategy led to the selection of a list of 117 variants that were thoroughly studied in order to select the candidate variants that can explain the clinical picture of the patient. One homozygous variant, the p.Tyr414* (c.1242C>A) in *XYLT2* (NM_022167.3), was considered as the only candidate variant based on the gene function. Sanger sequencing confirmed the presence of this mutation at a homozygous state in both affected patients (Figure 4) and at heterozygous state in the parents. The identified variant in the *XYLT2* gene is also absent in our local database and in gnomAD.

The selected variant is located in exon 6 of *XYLT2* and is predicted to be pathogenic by different pathogenic prediction tools [11]. It is classified as likely pathogenic (Class IV: PVS1 and PM2) based on the ACMG classification [11,12]. Indeed, the variant meets the criteria PVS1, since it is a nonsense variant in a gene where loss of function is a known mechanism causing a disease. It also meets the criteria PM2, because it is absent from control databases including our in-house database.

## 4. Discussion

In this paper, we described two Lebanese patients with SOS linked to a novel homozygous nonsense mutation in the *XYLT2* gene (p.Tyr414*). *XYLT2*, located on the long arm of chromosome 17q21.3–q22, encodes an 865 amino acid protein (NP_071450). As stated earlier, the protein transports xylose molecules from the nucleoside diphosphate donor (UDP–xylose) to targeted serine molecules of the core protein during the synthesis of PGs. XYLT2 comprises four different protein domains: an N terminal domain, a xylosyltransferase terminal domain, which is the catalytic domain of the enzyme, a core2/I-branching enzyme domain, and a C-terminal domain [13]. The herein identified nonsense variant is located in the Core-2/I-Branching enzyme domain. It can result in the expression of a short, truncated protein variant or in the activation of nonsense mediated decay, which may lead to the degradation of the protein. Unfortunately, functional studies were not possible to further evaluate the effect of the mutation on the protein structure and expression level.

To date, 22 additional patients with 12 different *XYLT2* mutations, including only one nonsense, six missense, two frameshift duplication, and three frameshift deletions, have been reported worldwide (Supplementary Table S1) [2,13,14,15,16,17,18].

A comparison between the clinical findings of all the reported cases (Supplementary Table S1) shows that the following phenotypic features appear to be the most prevalent: cataracts (24/24), long bone fractures (21/22), vertebral flattening (19/24), kyphosis (16/19), osteoporosis (16/21), short stature (15/23), facial dysmorphism (18/18), and hearing impairment (14/24). Intellectual disability (12/21), cardiac (8/24), and genitourinary (4/24) findings remain of variable prevalence. Life expectancy in patients with SOS syndrome seems to be normal.

Age of onset of the different clinical features seen in SOS is variable. It is worth mentioning that while in the three patients described by Munns et al. in 2015, cataract was considered a feature that develops in the second decade of life [2], based on the subsequently reported cases (Supplementary Table S1), cataract was shown to be present—as early as or even before 5 years of age, in some cases. This indicates that earlier ophthalmological evaluation is essential to prevent more vision complications in patients with SOS.

Due to the limited number of reported cases, it has been quite difficult to establish a genotype–phenotype correlation. That being said, several studies have suggested a possible association between the mutation type, localization, domain, and the severity of the clinical features. For instance, Taylan et al., 2016 attribute the presence of a nonsense mutation in one patient (Patient 4, Supplementary Table S1), to a severe presentation with underlying cardiac abnormalities that were absent in other patients with missense mutations [15]. Other reports have also discussed this hypothesis and suggested that frameshift mutations are associated with a more severe presentation compared to missense mutations [13,16]. Interestingly, a nonsense mutation was herein identified in both patients, with no cardiac, genitourinary, hearing impairment, nor hepatic/splenic anomalies and with cataracts being the most prominent eye finding. All together, these findings confirm the variable expressivity of this syndrome, rendering the correlation between the genotype and the phenotype hard to establish. This may also suggest the contribution of other genetic modifiers to the clinical phenotype. That being said, in the future, reporting additional cases with *XYLT2* mutations may help identify genotype–phenotype correlations and lead to clinical trials [19].

Pamidronate infusions (biphosphonates), extensively used for the treatment of some bone disorders, such as postmenopausal and glucocorticoid-induced osteoporosis, malignancy-induced hypercalcemia and Paget’s disease [20], have been shown to be effective for the treatment of patients with SOS [2,15]. Patient IV-4 has received bisphosphonates treatment through pamidronate infusions, which improved bone mineral density and fracture frequency without reshaping the compression fractures. Interestingly, this observation shows that this treatment may help reduce disease severity, but the mechanism of bisphosphonates therapy in SOS is yet to be discovered. Variable response to treatment was also reported by Kausar et al., which illustrates the complexity of the disease not only in terms of genetic mutations and phenotypic presentations, but also in terms of response to therapy [17]. Other observed clinical features such as developmental delay, cataract, cardiac malformations, and hearing impairment must eventually be treated accordingly.

SOS syndrome cannot be detected prenatally by ultrasonography. Doddato et al., 2021 report on one case that was diagnosed at birth with SOS but with prenatal findings on ultrasound that included increased nuchal translucency, hyperechoic intestine, oligohydramnios, and cystic hygromas [18]. Further reports on prenatal findings might be interesting. Meanwhile, if the mutation has been characterized in the family, prenatal diagnosis can be offered.

The clinical heterogeneity of SOS might render its diagnosis sometimes challenging. The differential diagnoses that could be initially considered are the syndromes belonging to the family of linkeropathies. They are characterized by a wide spectrum of manifestations including skeletal dysplasia, short stature, cutaneous anomalies, joint laxity, facial dysmorphism, heart malformation, and developmental delay [21]. To date, five genes, encoding various glycosyltransferases, have been linked to linkeropathies; besides the *XYLT2* gene, there are the genes *XYLT1* (OMIM * 608124), *B4GALT7* (OMIM * 604327), *B3GAT3* (OMIM * 606374), and *B3GALT6* (MIM:* 615291) [22]. *XYLT1* is linked to Desbuquois dysplasia 2 (OMIM # 615777), an autosomal recessive skeletal disorder characterized by growth retardation, joint laxity, short extremities, progressive scoliosis, and developmental delay, in the absence of hand abnormalities [23,24]. *B4GALT7* is involved in Ehlers–Danlos syndrome (EDS), spondylodysplastic type 1 (EDSSPD1, OMIM # 130070). EDS is a group of clinically and genetically heterogeneous heritable connective tissue disorders characterized by the triad of (generalized) joint hypermobility, cutaneous abnormalities, and internal organ/vascular fragility and dysfunctions [21]. *B3GAT3* (OMIM * 606374) is linked to multiple joint dislocations, short stature, and craniofacial dysmorphism, with or without congenital heart defects (OMIM # 245600). Patients with this disorder present with linkeropathy, including osteopenia with fractures (osteogenesis imperfecta-like), dislocations (Larsen-like), and developmental delay [25]. Finally, B3GALT6 (OMIM * 615291) is linked to the following three diseases: Al-Gazali syndrome (OMIM # 609465), Ehlers–Danlos syndrome, spondylodysplastic type 2 (EDSSPD2, OMIM # 615349), and spondyloepimetaphyseal dysplasia with joint laxity, type 1, with or without fractures (SEMDJL1; OMIM # 271640). Al-Gazali syndrome is characterized by skeletal anomalies, prenatal growth retardation including joint contractures, camptodactyly, and small mouth, eye anomalies, and early lethality [26]. Patients with EDSSPD2 present with short stature, craniofacial dysmorphism including an aged appearance, generalized osteopenia, muscles hypotonia, defective wound healing, loose skin, and developmental delay [27]. SEMDJL1 is characterized by skeletal and vertebral abnormalities, thoracic asymmetry, ligamentous laxity, progressive severe kyphosis, distinctive facial features, palatal abnormalities, and congenital heart disease [28]. All above listed syndromes or linkeropathies are characterized by the presence of fractures, vertebral and occular anomalies, short stature, developmental delay, and dysmorphic facial features as also described in SOS patients. However, what easily differentiates all of them from SOS is the absence of cataract, which is seen in 100% of patients presenting with SOS.

Further to linkeropathies, other possible differential diagnoses are the ones including multiple fractures associated with cataract. The first is the osteogenesis imperfecta-microcephaly cataract syndrome (OMIM # 259410), which can be ruled out by the presence of microcephaly and short limbs. Another differential diagnosis is the osteoporosis pseudoglioma syndrome (OMIM # 259770), which is an autosomal recessive disorder characterized by severe juvenile-onset osteoporosis and congenital or juvenile visual loss in patients associated with aberrant vitreo-retinal vascular growth. Fractures, cataract, short stature, intellectual disability, barrel chest, and kyphoscoliosis are part of the syndrome. It can be differentiated from SOS mainly by the presence of microcephaly and abnormal metaphyses and diaphyses of the long bones. Finally, there is the syndrome known as calvarial doughnut lesions with bone fragility with or without spondylometaphyseal dysplasia (OMIM # 126550) where fractures and cataract can be seen. It is simply differentiated from SOS by its different mode of inheritance and the presence of typical multiple hyperostotic or osteosclerotic lesions of calvarium.

Despite the distinctive features characterizing each of these entities, an accurate diagnosis remains challenging, requiring genetic testing for the confirmation of the clinical diagnosis and the orientation of the genetic counseling approach in the referred families. The implementation in the diagnostic path of WES and whole genome sequencing performed by Next Generation Sequencing (NGS) techniques have revolutionized the clinical genetics field through the discovery of new genes/disorders and the delineation of new clinical features in previously known disorders with variable expressivity. These approaches are considered today as effective diagnostic tools that are rapid and that overcome the challenges faced in the clinic, especially in the differential diagnosis process. In addition, and due to the continuous advances in the field of bioinformatics, these tools based on the latest NGS algorithms can now identify, with a certain confidence, the presence of small chromosomal aberrations such as deletions and duplications of more than one exon, thus replacing additional molecular techniques. Most importantly, and as already seen in many highly inbred populations where the prevalence of consanguineous marriages can exceed 50%, these diagnostic tools contribute widely to the field by pointing out the presence in the same family of two or more distinct entities initially mistaken as new syndromes [29]. Therefore, genetic testing and counseling should be rendered available at a large scale, especially in these populations that are prone to present orphan diseases. Advances in the molecular biology field have, for instance, enabled the implementation of neonatal screening for several treatable genetic diseases such as some inborn errors of metabolism. Furthermore, in families where the causative mutation have been identified, several genetic approaches can be useful, such as genome-wide noninvasive prenatal diagnosis of monogenic disorders (NIPD) [30] and preimplantation genetic diagnosis (PGD).

In this paper, we have reported the second nonsense mutation in *XYLT2* in patients with SOS, reviewed all previously reported cases, and contribute to a better delineation of the phenotypic spectrum of the disease.

## Data Availability

The datasets used and analyzed during the current study are available from the corresponding author upon reasonable request.

## Supplementary Materials

The following supporting information can be downloaded at: https://www.mdpi.com/article/10.3390/genes14020497/s1, Table S1: Clinical and genetic summary of all reported XYLT2-mutated cases.
